# Handling of lipemic samples in the clinical laboratory

**DOI:** 10.1515/almed-2023-0003

**Published:** 2023-02-20

**Authors:** Carla Fernández Prendes, María José Castro Castro, Lourdes Sánchez Navarro, Loreto Rapún Mas, Cristian Morales Indiano, Teresa Arrobas Velilla

**Affiliations:** Laboratory Medicine Department, Laboratori Clínic Metropolitana Nord, Hospital Universitari Germans Trias I Pujol, Badalona, Spain; Laboratory of Nutrition and Cardiovascular Risk, Hospital Universitario Virgen Macarena, Sevilla, Spain; Workgroup of Lipoproteins and Cardiovascular Diseases, Spanish Society of Laboratory Medicine, Barcelona, Spain; Biochemistry Core, Laboratori Clínic Territorial Metropolitana Sud, Hospital Universitari de Bellvitge, L’Hospitalet de Llobregat, Spain; Haematological Core, Laboratori Clínic Territorial Metropolitana Sud, Hospital Universitari de Bellvitge, L’Hospitalet de Llobregat, Spain

**Keywords:** interference, intralipid, lipemia, lipemia index, serum indices

## Abstract

Interferences in the clinical laboratory may lead physicians misinterpret results for some biological analytes. The most common analytical interferences in the clinical laboratory include hemolysis, icterus and lipemia. Lipemia is defined as turbidity in a sample caused by the accumulation of lipoproteins, mainly very-low density lipoproteins (VLDL) and chylomicrons. Several methods are available for the detection of lipemic samples, including the lipemic index, or triglyceride quantification in serum or plasma samples, or mean corpuscular hemoglobin (MCHC) concentration in blood samples. According to the European Directive 98/79/CE, it is the responsibility of clinical laboratories to monitor the presence of interfering substances that may affect the measurement of an analyte. There is an urgent need to standardize interference studies and the way interferences are reported by manufacturers. Several methods are currently available to remove interference from lipemia and enable accurate measurement of biological quantities. The clinical laboratory should establish a protocol for the handling of lipemic samples according to the biological quantity to be tested.

## Introduction

Analytical interferences in the laboratory cause significant abnormalities in biological quantities. These abnormalities may be clinically significant, leading to the misinterpretation of results.

One of the most common analytical interferences in the clinical laboratory is lipemia [1]. Lipemia is defined as turbidity in a sample caused by the accumulation of lipoproteins, mainly very-low density lipoproteins (VLDL) and chylomicrons. These particles are rich in triglycerides.

Lipoproteins exhibit a high heterogeneity in size and not all contribute equally to turbidity. Chylomicrons are the largest lipoprotein particles (70-1000 nm) and cause the most turbidity in the sample. Based on size, there are three types of very low density lipoproteins (VLDL): small (27–35 nm); medium (35–60 nm); and large (60–200 nm). Only large and medium VLDL cause turbidity. High-density lipoproteins (HDL) (6–12.5 nm) and low-density lipoproteins (LDL) (20–26 nm) do not cause turbidity [2, 3].

The frequency of lipemic samples is 0.5–2.5%, depending on the characteristics of the hospital and inpatient-to-outpatient ratio. Hemolysis increases when erythrocytes are suspended in lipemic plasma; therefore, its frequency increases as lipemia augments [4].

The most common pre-analytic cause of lipemia is short fasting time. However, recent studies demonstrate that non-fasting status does not induce significant changes in lipoprotein concentrations. Thus, fasting is indicated when non-fasting triglycerides are >400 mg/dL (4.56 mmol/L) [5].

Severe lipemia causing significant interference may occur in primary (familial chylomicronemia syndrome) or secondary (diabetes mellitus, insulin resistance, alcoholism, human immunodeficiency virus infection, kidney disease, among other) hypertriglyceridaemias [1]. Parenteral nutrition and diluents for poorly water-soluble medications containing lipidic emulsions may also cause lipemia [6].

## Scope

This document provides information about lipemia interference in the measurement of biological quantities and propose a standard protocol for the management of lipemic samples in the laboratory.

## Interference from lipemia

Analytical interference is defined as a significant change in the measurement of a biological quantity due to the presence of an interfering substance in the sample.

Interference is investigated by comparing the assay under study against an assay not affected by any interference. Currently, assays for most of biochemical quantities are susceptible to lipemia. Therefore, assays are performed using different procedures: by adding synthetic lipids mimicking the turbidity of native lipids [7] or by removing lipids from lipemic patient samples by ultracentrifugation [8].

According to the European Directive 98/79/CE, it is the responsibility of reagent manufacturers to investigate interferences and report a maximum permissible error [9].

Most manufacturers use Intralipid^®^ to simulate lipemia in serum. Intralipid^®^ is a lipid emulsion for intravenous perfusion containing purified soybean oil, egg yolk phospholipids and glycerin. Interference is considered to be significant when the result for a biological quantity before and after adding a specific concentration of synthetic lipids has a bias of 10% [10].

The interference studies conducted by manufacturers have some limitations: (a) the biological variability of quantities is not considered; (b) the composition of synthetic lipids differs from that of endogenous lipids, resulting in inconsistent results between lipemic samples and Intralipid^®^-supplemented serum samples [10], [11], [12]; (c) there are no standard materials that mimic lipemia correctly; and (d) if the composition of synthetic lipids contains glycerol, it interferes with triglycerides, since the automated analyzers used for testing triglycerides do not perform a glycerol blank test [13].

Moreover, the maximum permissible error reported by manufacturers varies with the instrument, analytical method, and criterion used. Roche diagnostics and Beckman Coulter analyze lipemia interference by adding Intralipid^®^ to serum samples, but use different reporting criteria (Table 1).

**Table 1:** Interferences from lipemia in a variety of biological analytes reported by two manufacturers, Roche diagnostics and Beckman coulter.

Biological analytes	Roche diagnostics	Beckman coulter
	Intralipid^®^, mg/dL	
Srm—Alanine aminotransferase; cat.c.	150	300
Srm—Albumin; mass c.	550	800
Srm—Aspartate aminotransferase; cat.c.	150	300
Srm—alpha-Amylase; cat.c.	1,500	1,000
Srm—Apolipoprotein A-I; mass c.	1,000	1,000
Srm—Bilirubin; subst.c.	1,000	1,000
Srm—Bilirubin (esterified); subst.c.	750	1,000
Srm—Calcium (II); subst.c.	1,000	1,000
Srm—Chloride; subst.c.	2,000	500
Srm—Cholesterol; subst.c.	2,000	1,000
Srm—Cholesterol-HDL; subst.c.	2,000	900
Srm—Creatinine; subst.c.	2,000	1,000
Srm—Creatine-kinase; cat.c.	1,000	1,000
Srm—Rheumatoid factors; arb.subst.c. (OMS 64/2)	2,000	750
Srm—Ferritin; mass c.	1,000	1,000
Srm—Alkaline phosphatase; cat.c.	2,000	1,000
Srm—Phosphate; subst.c.	800	800
Srm—gamma-Glutamyltransferase; cat.c.	2,000	1,000
Srm—Glucose; subst.c.	1,000	700
Srm—Haptoglobin; mass c.	600	1,000
Srm—Iron (II + III); subst.c.	1,500	100
Srm—Immunoglobulin A; mass c.	2,000	1,000
Srm—Immunoglobulin G; mass c.	2,000	1,000
Srm—Immunoglobulin M; mass c.	2,000	200
Srm—Lithium; subst.c.	2,000	2,000
Srm—Magnesium (II); subst.c.	2,000	200
Srm—Potassium; subst.c.	2,000	500
Srm—Protein; mass c.	200	1,000
Srm—C-reactive protein; mass c.	1,000	1,000
Srm—Sodium; subst.c.	150	500
Srm—Transferrin; subst.c.	500	1,000
Srm—Urea; subst.c.	1,000	500
Srm—Urate; subst.c.	1,500	1,000

Ultracentrifugation for the removal of lipids in lipemic samples also has some limitations. As lipoproteins in lipemic samples vary in size and amount, deviation caused by lipemia in biological quantities is uncertain. Thus, further studies based on larger representative samples are necessary [14].

There is an urgent need to standardize the procedures by which manufacturers investigate and report lipemia interference. Clinical laboratories should also be aware of the potential lack of replicability [15].

Reagent manufacturers do not perform interference tests in biological fluids due to the large variety of matrices available. Serous fluids, however, may contain high concentrations of lipids that cause turbidity.

Biochemical analysis of biological fluids is clinically relevant in some settings. Light’s criteria compare total protein concentrations in pleural fluid and lactate dehydrogenase against serum concentrations to differentiate exudative from transudative effusion, which provides diagnostic information. Estimation of albumin gradient in peritoneal effusion is based on the determination of albumin in ascites and serum to determine whether ascites is caused by portal hypertension. Other clinically relevant body fluid tests include determination of cholesterol and triglycerides in pleural fluid to detect chylothorax and pseudochylothorax [16]; glucose for infectious or malignant effusions; and amylase for the detection of postoperative pancreatic fistulas.

## Mechanism of interference from lipemia

Three mechanisms are involved in lipemia interference: light scattering, volume displacement effect, and lack of sample homogeneization [10].

### Light scattering by lipoproteins

The main mechanism of lipemia interference with some quantities is light scattering caused by lipoproteins (mainly chylomicrons and VLDL). Lipemia causes light scattering across the visual spectrum (300–700 nm) and increases as wavelength decreases. Colorimetric assays taking absorbance readings at the shorter wavelengths of the visual spectrum are most susceptible to interference [10]. The sign and extend of interference in spectrophotometric methods depends on whether the method measures an increase or a decrease of absorbance, and the wavelength used. For that reason, lipemia interference may not be comparable between two analytical methods (Table 1). This mechanism affects spectrophotometric, nephelometric and turbidimetric assays.

The size and composition of lipoproteins influence light scattering. Both, chylomicrons and VLDL are very heterogeneous in size, thereby contributing to sample turbidity differently [2, 3].

### Volume displacement effect

This mechanism affects sodium determination strongly. It also affects other electrolytes, including potassium and chloride, although interference is rarely clinically significant.

Most autoanalyzers measure electrolyte concentrations by indirect potentiometry using ion-selective electrodes and a sample dilution of 1:20 to 1:34. The result is obtained from a calculation based on the aqueous composition of the serum or plasma matrix.

Serum and plasma are composed of 92% aqueous phase and 7% solid phase. Both, in lipemic samples and in samples of patients with hyperproteinemia, the aqueous phase decreases, thereby resulting in an underestimation of the constituents that distribute in this phase, such as electrolytes. This effect is known as “volume displacement effect” [10, 17], [18], [19].

### Lack of sample homogeneization

This effect results from the difference in density of particles in serum or plasma samples. After centrifugation, chylomicrons and VLDL will be located at the top of the tube due to their low density, whereas the remaining constituents will distribute depending on their polarity. Thus, hydrophobic components distribute in the lipid phase (upper part of the tube) whereas hydrophilic components distribute in the aqueous phase (lower part of the tube).

Analyzers use sensors to prevent the needle from going too deep into the tube to obtain a sample from the upper part of the tube. This may result in a falsely decreased concentration of hidrophobic electrolytes. The opposite occurs with hydrophic substances (valproic acid or steroid hormones), which accumulate in the upper lipid layer [2, 3, 10].

### Other lipemia interference mechanisms

The accumulation of lipoproteins in serum may interfere with biological quantities measured based on physical and chemical interactions. This is especially relevant in electrophoretic methods [20].

Lipemia may also interfere in some immunoassays, since lipoproteins may interfere with antigen-antibody reaction by blocking the binding sites of antibodies [21].

Likewise, lipemia may cause abnormalities in neutrophils including increased size, degranulation, and cytoplasmic vacuolization [22]. Elevation of triglycerides may modulate neutrophil signaling and increase expression, adhesion and permeability of the membrane [23]. These morphological changes may interfere in neutrophil identification by analyzers that use light scatter to characterize white blood cell differential count. Lipemia causes the enlargement of neutrophils, which may overlap with monocytes on WBC scattergram. Lipemia may cause abnormalities in erythrocyte margins, which appear blurred in blood smear. This finding is useful when hypertriglyceridemia is not suspected and further laboratory results are not available.

Based on the type of lipoprotein in the sample, total WBC and platelet count may be falsely elevated. This interference occurs both in analyzers that determine leukocyte and platelet count by impedance and by optic methods, being interference more pronounced in the latter. This is explained by the fact that lipoproteins have a high refraction index and generate abnormal signals in platelet and/or leukocyte channels. In addition, hemoglobin concentrations measured by spectrophotometry in hematological analyzers may yield falsely elevated concentrations when triglycerides are elevated [24].

## Lipemia index measurement methods

In patient samples, lipemia is most often detected by visual inspection, especially in laboratories that work with a low number of samples. However, this method is subjective, arbitrary and not sensitive enough. Turbidity caused by lipemia in serum or plasma is often detected visually when triglycerides are >300 mg/dL (3.42 mmol/L) [25]. Visual detection in whole blood can only be performed if cells are separated by sedimentation or centrifugation.

An alternative approach to visual detection, although with some limitations, is measuring concentrations of triglycerides based on the degree of lipemia or turbidity. The degree of turbidity does not correlate with triglyceride concentration, since the proportion of triglycerides differ according to the type of lipoprotein in the sample [10, 26].

Automated serum index measurement is currently used in routine biochemical analysis. Serum indices measure the presence of potential interferents: hemoglobin, bilirubin and turbidity (mainly due to lipemia).

Serum indices are measured by diluting the sample in a saline or buffer solution. Absorbance readings provide a semi-quantitative calculation of the degree of icterus, hemolysis or lipemia in serum/plasma. Some methods use a sodium chloride (NaCl) solution at 0.9% as reagent and yield absorbance readings at different wavelengths, which enables lipemia index calculation. Formulas include corrections to overlapping spectra [27].

Lipoproteins scatter light around 700 nm; therefore, these wavelengths are used to assess the degree of lipemia. Although manufacturers use different wavelengths, almost all use combinations of two or more wavelengths. For example, on the AU series (Olympus), Beckman Coulter uses 660/800, on Cobas series, Roche uses 660/700 and on Architect, Abbott uses several wavelengths (510/524; 572/604; 628/660 and 524/804) in a calculation of the degree of lipemia [28]. In the case of Werfen analyzers, ACLTOP series 50, lipemia interference is assessed by measuring optic absorbance of the sample diluted at three different wavelengths (405, 535 and 671).

Lipemia index is measured in lipemia units, which are linear until 2,000 mg/dL (22.8 mmol/L) and is based in the optic behavior of Intralipid^®^. However, Intralipid^®^ composition differs from that of natural lipids [10, 27].

The automatic detection of lipemia has multiple advantages: low cost, high speed, increased reproducibility and shortening of turnaround time. This method, however, also has some disadvantages, such as falsely elevated lipemia indices in samples with increased turbidity caused by the presence of other molecules such as paraproteins [29, 30].

When assessing homeostasis, some analyzers use a pre-analytical module that detects lipemia. Intervals over which lipemia interference occurs are established by manufacturers. When the lipemia index exceeds the threshold established for each quantity, the analyzer will trigger an alarm. Some analyzers that do not have a pre-analytical module initiate an alarm of possible lipemia. Then, the sample is visually inspected.

In whole blood, there are no indices available that indicate sample quality. Lipemia can be hardly detected in whole blood. In this case, mean corpuscular hemoglobin concentration (MCHC) is the most sensitive index for the detection of lipemia. It is estimated that MCHC >360 g/L (>36 g/dL), after other potential causes such as hemolytic anemia or agglutinins have been excluded, may be caused by lipemia, as it induces an increase in hemoglobin concentration [24].

Hemoglobin is measured by spectrophotometry (generally determined at 425 nm) and turbidity induced by lipemia causes falsely elevated concentrations. As hemoglobin concentration is erroneous, erythrocyte indices, which are calculated from hemoglobin, are also erroneous. This results in falsely elevated mean corpuscular hemoglobin (MCH) and mean corpuscular hemoglobin (MCHC).

As an alternative to hemoglobin quantification, some blood analyzers evaluate laser dispersion in the reticulocyte channel. The blood autoanalyzers that use the two methods initiate an alarm of possible lipemia interference when they detect differences in hemoglobin concentration measured by photometry and by optical dispersion. These analyzers also use the difference between MCHC (hemoglobin measured by photometry) and estimated MCHC (hemoglobin measured by optical dispersion) to detect interference. A limitation of these quantities is that they are not reportable. Some graphs, such as leukocyte histograms based on the impedance method, dispersion plots of leukocyte subpopulations (fluorescence, laser dispersion) or erythroblasts graphs (laser dispersion) may provide an alarm of lipemia.

## Lipemia removal methods

### Centrifugation methods

Serum/plasma centrifugation is the method of choice for removing lipemia. Sample ultracentrifugation (100,000–2,000,000×*g*) removes lipids effectively and facilitates analyte determination [31, 32]. Ultracentrifugation requires using equipment that is not available in the majority of laboratories. However, large lipoproteins (chylomicrons) can be separated in serum/plasma by high-speed centrifugation (10,000–15,000×*g*) [33, 34]. When lipemia is caused by VLDL accumulation, the procedure is less effective.

After high-speed centrifugation, a lipid layer forms in the upper part, and the infranatant is collected using a glass pipette with care not to contaminate the sample with the lipid layer. Biochemical quantities are measured in the infranatant. This method is not appropriate for hydrophobic substances (hormones and drugs, among others), since they would distribute across the lipid layer and measurement in the infranatant would give falsely low results.

### Extraction methods

Removal of lipids from serum/plasma is performed using polar solvents such as 1,1,2-trichlorotrifluoroethane.

Lipoclear^®^ is a non-ionic polymer that has been extensively used in the laboratory to remove lipemic samples. However, it is not currently available in the market.

The addition of an exogenous substance may cause interference in the measurement of biochemical quantities. It is the case of proteins, calcium, and aspartate aminotransferase when the sample is treated with 1,1,2-trichlorotrifluoroethane; or the case of proteins, albumin, and calcium when samples are treated with Lipoclear^®^ [35].

### Dilution or replacement methods

Concentrations of an interferent can be reduced or eliminated through serum/plasma dilution, especially in lipophilic components with significant diffusion in the lipid phase. The sample should be diluted to reduce lipemia below the limit of interference of the relevant quantities, but not too much to make sure that component concentration remains within the limit of quantification of the quantities [2].

Replacing plasma with the same volume of isoosmotic dilutant is a good strategy to reduce interference in blood samples. Nevertheless, results may be misleading, since cell loss may occur during replacement.

### Alternative measurement methods

Some gasmeter analyzers perform direct potentiometric or amperometric analysis to measure ions or creatinine, respectively. Hemostatic quantities such as prothrombin time or activated partial thromboplastin clotting time can be determined by electromechanical clot detection methods. In some analyzers, hemoglobin concentration is calculated from an algorithm based on erytrocyte concentrations measured by flow cytometry. However, the use of this technology in the clinic has not been approved.

## Lipemic sample detection and treatment protocol

Different strategies are used in Europe for the detection and treatment of lipemic samples. A standard protocol for the handling of lipemic samples in the clinical laboratory has not yet been established in Europe. The European Federation of Clinical Chemistry and Laboratory Medicine (EFLM) Working Group for the Preanalytical Phase conducted a survey among 1,416 laboratories from 45 European countries on the handling of lipemic samples to collect the data necessary to provide recommendations and standardize processes.

This survey revealed that 14% of laboratories do not assess lipemia in samples. Measurement of lipemia was performed automatically by lipemia indices in 43% (n=493) of laboratories; by visual inspection of samples in 30% (n=348); and by a combination of automatic and visual methods in 28% (n=319) of the laboratories that measured lipemia. Only 25% (n=203) verified the quality of measurements using internal quality controls. Thirty-seven percent of laboratories performed additional measurements of triglycerides in lipemic samples. Only 27% of laboratories used plasma delipidation methods, being centrifugation the most frequent method, followed by dilution or use of specific reagents. In total, 72% of laboratories rejected only those quantities affected by lipemia and included a comment; 21% released all test results and reported the presence of lipemia; 6.6% released all results without including any comment on the report [36].

**Figure 1:**
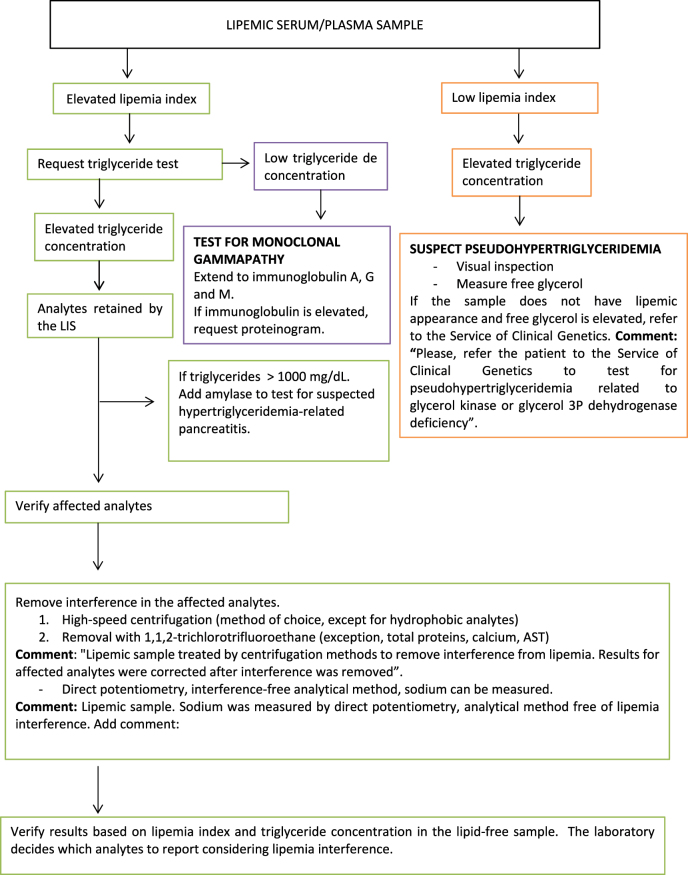
Flowchart for the detection and treatment of lipemic serum/plasma detection in the analysis of biological analytes.

**Figure 2:**
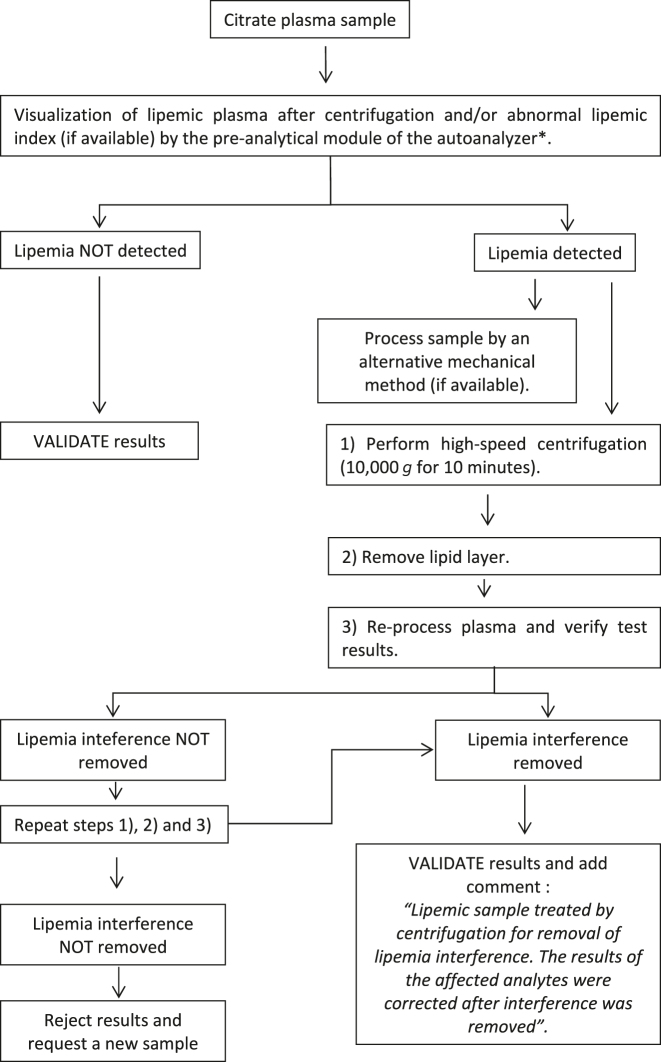
Flow chart for the detection and treatment of lipemic citrate plasma samples for the analysis of hemostatic analytes. *Method available in some hemostasis analyzers.

**Figure 3:**
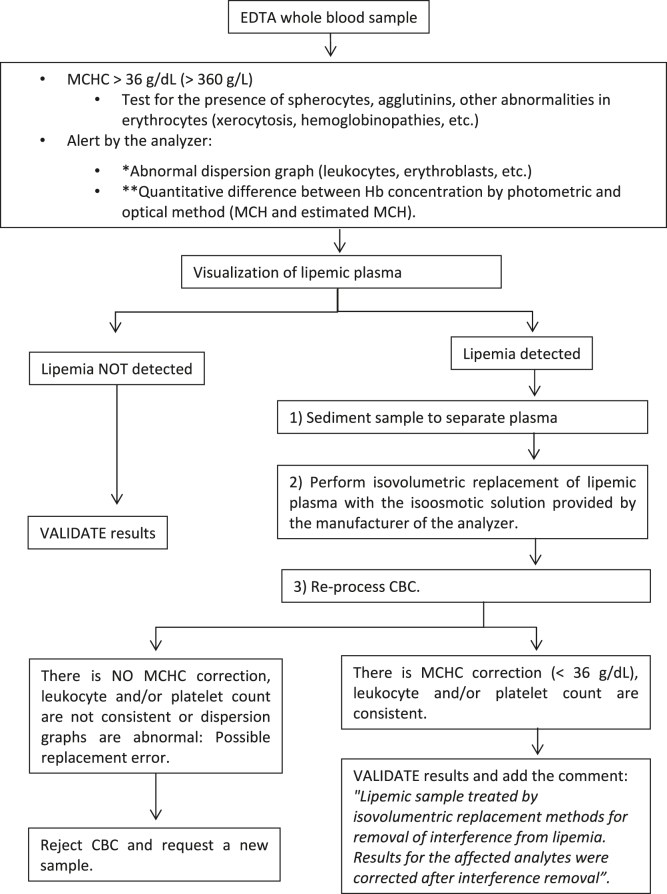
Flow chart for the detection and treatment of lipemic EDTA whole blood samples for the analysis of CBC analytes. CBC, complete blood count; MCHC, mean corpuscular hemoglobin concentration; MCH, mean corpuscular hemoglobin; Hb, hemoglobin. *The abnormal graph is specific of each hematology analyzer. **Only available in some hematology analyzers.

Based on the results of this European survey, we propose several protocols for the detection and handling of lipemic samples for the harmonization of processes. Three protocols are suggested with their corresponding flowcharts based on the types of samples and quantities to be measured: quantities in serum or plasma (Figure 1); quantities of hemostasis in plasma (Figure 2); and quantities of complete blood count in whole blood (Figure 3).

### Biochemical quantities in serum or plasma

Quantities affected by lipemia should be retained during validation by the laboratory information system (LIS) using an algorithm based on lipemia index. When lipemia index is high, testing should be extended to triglycerides using the automated LIS rule.

The limitations of the two methods make it necessary that they are both used. The lipemia index is based on the optic behavior of Intralipid^®^, and triglyceride concentration does not correlate with the degree of turbidity, since turbidity varies with the type of liprotein in the sample [26], [27], [28].

When a result is retained by the LIS, verification should be as follows:

– If interferences were not investigated in patient lipemic samples (endogenous lipids), check the quantities that may be affected, as reported by the manufacturer (Table 1).
– Subject the sample to high-speed centrifugation (10,000 g, 10 min) and reprocessing, which is the method of choice for removing lipemia interference [33, 34]. Include a comment on the report: “*Lipemic sample treated by centrifugation methods to remove interference from lipemia. Results for affected quantities were corrected after interference was removed*”.
– Repeat measurement of lipemia and triglycerides. If interference persists, remove lipemia using solvents such as 1,1,2-trichlorotrifluoroethane [34, 35], reprocess the sample treated and add the comment “*Lipemic sample treated by extraction methods for elimination of interference from lipemia. Results for the affected quantities were corrected after interference was removed*”.
– Repeat measurement of lipemia and triglycerides. The laboratory will determine the test results to be reported considering the final lipemic interference.
– Measure sodium concentration using a blood gasometer analyzer that utilize direct ion selective electrodes [19] and add the comment “*Lipemic sample. Sodium was measured by direct potentiometry, a lipemia-free laboratory method*”.
– In patients with triglycerides >1,000 mg/dL (11.4 mmol/L), which is suggestive of hypertriglyceridemia-related pancreatitis [37], amylase concentration in serum may also be added.
– A high triglyceride concentration and a low lipemia index are suggestive of pseudohypertriglyceridemia secondary to elevated basal glycerol. In this context, inspect the sample visually and measure free glycerol. If the sample does not seem lipemic on visual inspection, referral for genetic testing is recommended. Add a comment on the report: “*Please, refer the patient to the Service of Clinical Genetics for suspected pseudohypertriglyceridemia related to glycerol kinase or glycerol 3P dehydrogenase deficiency*”. Glycerol presents positive interferences with triglycerides because most reagents do not perform a glycerol blank test [38, 39].
– A high lipemia index accompanied by a normal/low triglyceride concentration indicates that the sample is likely to contain paraproteins. The addition of immunoglobulin A, G and M is recommended. If one of these immunoglobulins is high, a proteinogram can be added to exclude monoclonal gammapathies [40].

### Hemostasiologic quantities in plasma

In the presence of lipemia in a sample received for coagulation testing, the following processes are recommended by order of priority [41]:

– Visual inspection of the lipemic sample after centrifugation or, in case the analyzer includes a preanaltyical module, verify the lipemia index.
– Repeat measurement by an alternative mechanical method.
– If a mechanical method is not available, perform high-speed centrifugation (10,000×*g*, 10 min). Remove lipid layer and reprocess plasma.
– If interference from lipemia cannot be removed, repeat high-speed centrifugation (10,000×*g*, 10 min).
– Once interference has been removed, validate results for hemostasis quantities and add the comment: “*Lipemic sample treated by centrifugation for removal of interference from lipemia. The results for the affected quantities were corrected after interference was removed*”.

### Hematimetric quantities

In complete blood counts with MCHC >36 g/dL possibly due to lipemia interference, the following processes are recommended:

Check for the presence of spherocytes (suggestive of hemolytic anemia) by visual inspection of peripheral blood smear. Add comment: “*Measuring reticulocytes and biochemical hemolysis quantities (haptoglobin, bilirubin, lactate dehydrogenase) completes diagnostic studies of hemolytic anemia*”.

Visual inspection of the sample following sedimentation.

If the sample does not have lipemic appearance (and there is no suspicion of hemolysis spherocytes), heat the sample (37 °C, 30 min) and reprocess. If interference does not disappear, extend time to 1 h or even 90 min. This effect may be due to the presence of agglutinins in the sample.

If the sample seems lipemic, follow the process below:

Perform isovolumetric replacement of plasma with the isoosmotic dilutant provided by the manufacturer of the analyzer and re-evaluate complete blood count. If MCHC <36 g/dL, verify that isovolumentric replacement was performed correctly by checking that leukocyte and platelet counts are consistent with the ones obtained in the original sample. The other quantities and graphs provided by the analyzer should not show abnormalities. Validate results by adding the comment: “*Lipemic sample treated by isovolumentric replacement methods for removal of interference from lipemia. Results for the affected quantities were corrected after interference was removed*”.

If after isovolumentric replacement of plasma complete blood count results are not appropriate (possible replacement error), reject the complete blood count, report rejection by adding a comment, and request a new sample.

## Conclusions

A review was performed of the different mechanisms of lipemia interference and diverse strategies used to prevent it. This review is intended to serve as a guide for clinical laboratory professionals.

It is important that lipemia index in serum/plasma and triglyceride concentrations are determined using a semi-quantitative method. Potential interferences in the measurement of triglycerides should always be considered. The presence of elevated concentrations of triglycerides and low lipemia indices should raise suspicion of pseudohypertriglyceridemia due to elevated basal glycerol.

Determination of serum indices has some limitations. Elevated serum indices and low levels of triglycerides indicate the potential presence of paraproteins.

High-speed centrifugation can be used as the method of choice for removing serum/plasma samples and measure biochemical, non-lipohphilic components or hemostasiologic quantities. As a second choice for serum/plasma samples, remove lipids using polar solvents (1,1,2-trichlorotrifluoroethane).

For lipohilic components measured in plasma/serum, sample dilution is recommended. For hematologic quantities in whole blood, isovolumetric replacement with an isoosmotic dilutant is recommended.
